# The study protocol for a non-randomized controlled clinical trial using a genotype-guided strategy in a dataset of patients who undergone percutaneous coronary intervention with stent

**DOI:** 10.1016/j.dib.2016.12.019

**Published:** 2016-12-20

**Authors:** Cristina Lucía Dávila-Fajardo, Jesús Sánchez-Ramos, Xando Diaz- Villamarín, Luis Javier Martínez-González, Pablo Toledo Frías, Susana Martínez Huertas, Francisco Burillo Gómez, Juan Caballero Borrego, Alicia Bautista Pavés, Mª Carmen Marín Guzmán, José Antonio Ramirez Hernández, Concepción Correa Vilches, Jose Cabeza Barrera

**Affiliations:** a Department of Clinical Pharmacy, Granada University Hospital, Institute for Biomedical Research, ibs., Granada, Spain; b Department of Cardiology, Granada University Hospital, Institute for Biomedical Research, ibs., Granada, Spain; c Genomics Unit, Centre for Genomics and Oncological Research (GENYO), Pfizer-University of Granada-Andalusian Regional Government, Health Sciences Technology Park, Granada, Spain

**Keywords:** Clinical trial protocol

## Abstract

This article contains data related to the research article entitled “Results of genotype–guided antiplatelet therapy in patients undergone percutaneous coronary intervention with stent” (J. Sánchez-Ramos, C.L. Dávila-Fajardo, P. Toledo Frías, X. Díaz Villamarín, L.J. Martínez-González, S. Martínez Huertas, F. Burillo Gómez, J. Caballero Borrego, A. Bautista Pavés, M.C. Marín Guzmán, J.A. Ramirez Hernández, C. Correa Vilches, J. Cabeza Barrera, 2016) (1). This data article reports, for the first time, about the non-randomized clinical trial protocol that check if *CYP2C19/ABCB1* genotype–guided strategy in which the choice of antiplatelet therapy is based on the genetic test, reduces the rates of cardiovascular events and bleeding compared to a non-tailored strategy in patients undergone percutaneous coronary intervention (PCI) with stent. The data included in this article are: design and setting of the study, study population, inclusion and exclusion criteria, definition of the intervention, objectives, variables (baseline characteristics and during the follow-up), study procedures, collection and treatment of the biological sample, genotyping, withdrawal criteria, sample size, statistic analysis, ethical aspects, information sheet and consent form. The authors confirm that this study has been registered in Eudra CT (Eudra CT: 2016-001294-33).

**Specifications Table**

**Table:** 

Subject area	*Pharmacogenetics*
More specific subject area	*Cardiovascular diseases*
Type of data	*Figure and text file*
How data was acquired	*electronic health record system, access database, saliva sample*
Data format	*first as a draw data, then processed and then analyzed*
Experimental factors	*DNA genotyped using Taqman*^®^*allelic discrimination*
Experimental features	*data were analyzed using statistical software R*
Data source location	*Granada, Spain*
Data accessibility	*Data is within this article*

**Value of the data**

• In this study we describe the protocol we use for implementing a therapeutic scheme in which the genetic profile of each patient is contemplated using a personalized antiplatelet drug therapy. The protocol describes the design, study population, inclusion and exclusion criteria, objectives, variables, study procedures, collection data, genotyping, withdrawal criteria, sample size, statistic analysis, ethical aspect, information brochure patient and consent form.
• This protocol could be used as a model to achieve other clinical trials where the choice of antiplatelet therapy based on the genetic test is analyzed.
• Being a new study, it is important that future studies follow a similar protocol so that the results can be comparable. This is important since the evidence is sometimes not reproducible because the lack of homogeneity in the design of studies.

## 1 Data

The dataset of this article is the protocol achieved in Sánchez-Ramos et al. [1]

## 2 Experimental design, materials and methods

### 2.1 Designing and setting

This study is designed as an experimental non-randomized, single-center trial to check if *CYP2C19/ABCB1* genotype–guided strategy, in which the choice of antiplatelet therapy based on the genetic test, reduces the rates of cardiovascular events and bleeding compared to a non-tailored strategy.

### 2.2 Study population

The target population is patients with coronary artery disease, most of them with acute coronary syndrome (ACS), undergoing PCI with stent and indicating antiplatelet therapy (prasugrel / clopidogrel/ticagrelor was approved during the course of the study)) recruited in San Cecilio University Hospital, Granada, Spain. The follow-up period will be 12 months. The recruitment period started in April 2010 and is scheduled to end in September 2013. The duration of the study is 3 years including patient recruitment and assessment of the results.

### 2.3 Inclusion and exclusion criteria

– Inclusion:

• Patient greater than or equal to 18 years.
• To sign the consent to participate in the study.
• With diagnosis of coronary artery disease undergone PCI with stent
• Treated with the study medication (clopidogrel / prasugrel/ticagrelor) for 1 to 12 months

– Exclusion: patients requiring treatment with oral anticoagulation, presenting contraindication to take acetylsalicylic acid (ASA), clopidogrel, prasugrel or ticagrelor, or high risk of bleeding

### 2.4 Definition of the intervention

Once included in the study, all patients receive standard treatment required for coronary artery disease prior to PCI and stent implantation, including ASA 100 mg/day indefinitely. Within the group of genotyping, patients who carry *CYP2C19* loss of function alleles and/or wrong transporter *ABCB*1 3435 (TT) genotypes receive prasugrel (5 or 10 mg daily according to weight and age) or ticagrelor (90 mg twice daily) as a second antiplatelet therapy, and the remaining patients with the normal function in *CYP2C19* and *ABCB1* genes receive clopidogrel at doses of 75 mg/day, both during 1–12 months. In the control group patients are treated mainly with clopidogrel 75 mg/day (Supplementary Fig. 1).

### 2.5 Objectives

#### 2.5.1 Primary objective

1. To evaluate the usefulness of *CYP2C19* and *ABCB1* genotype–guided strategy (screening) in the choice of drug treatment of patients with coronary artery disease undergoing PCI with stent, ie, if the genotyping increases the effectiveness of clopidogrel. To compare the efficacy of clopidogrel: Genotyping (clopidogrel + prasugrel+ ticagrelor) and not genotyping (clopidogrel).
2. To compare the efficacy of clopidogrel, prasugrel and ticagrelor, considering the possible modifying effect of the polymorphism of *CYP2C19* and *ABCB1* genes on the response to clopidogrel (in the choice of drug treatment) in patients undergoing PCI and stent in which antiplatelet therapy platelet is indicated. To compare the efficacy of antiplatelet drugs in genotyping arm.

#### 2.5.2 Secondary objective

– To determine the genotypes in the control population and study population. To compare allelic frequencies.
– To compare the safety of clopidogrel in genotyping group and in control group.
– Compare the safety of clopidogrel, prasugrel and ticagrelor, clopidogrel considering gene interaction in the choice of drug treatment.

### 2.6 Assessment variables

#### 2.6.1 Baseline characteristics

*Independent variables related to baseline patient characteristics:*

– Age,
– Index body mass,
– Race / ethnicity (Caucasian, Asian, black…)
– Risk-factors: family history of coronary artery disease (yes / no), consumption of tobacco (yes / no), alcohol consumption, previous heart disease (yes / no), dyslipidemia (yes / no), hypertension (yes/no), diabetes (yes / no).

*Independent variables related to the pathology:*

– ST-elevation myocardial infarction (STEMI), non-STEMI, stable angina.
– Number of branches implanted with stent
– Revascularization: PCI (normal stent, drug eluting stent)
– Characteristics related to treatment: duration and concomitant drugs: statins, beta-blockers, ACE inhibitors, calcium antagonists, vitamin K antagonists, proton pump inhibitors
– Time of appearance of an event from the start of treatment with clopidogrel/prasugrel/ticagrelor.

#### 2.6.2 Variables to be collected during follow-up

*Effectiveness variables*

##### 2.6.2.1 Primary

The combined cardiovascular death, nonfatal ACS or nonfatal stroke defined as:

– Cardiovascular death: death from any demonstrable cardiovascular cause or death not clearly attributable to a non-cardiovascular causes.

ACS: refers to any group of clinical symptoms compatible with acute myocardial ischemia and covers the spectrum of clinical conditions ranging from unstable angina (UA) to non—ST-segment elevation myocardial infarction (NSTEMI) to ST-segment elevation myocardial infarction (STEMI).

STEMI: ischemic symptoms and persistent electrocardiogram ST segment elevation and positive troponin

NSTEMI: ischemic symptoms but no persistent ST-segment elevation and positive troponin

UA: no such biomarker can be detected in the bloodstream hours after the initial onset of ischemic chest pain. UA exhibits 1 or more of 3 principal presentations: [1] rest angina (usually lasting >20 minutes), [2] new-onset (<2 months previously) severe angina, and [3] a crescendo pattern of occurrence (increasing in intensity, duration, frequency, or any combination of these factors).

– Non fatal stroke: neurological deficit of 24 hours or more with confirmation by Computed Tomography (CT) or Magnetic Resonance Imaging (MRI).

##### 2.6.2.2 Secondary variables

– Urgent target vessel revascularization, defined stent thrombosis or coronary ischemic event not related to stent thrombosis. Number of branches implanted with stent (normal stent, drug eluting stent)

*Safety*: TIMI major and minor bleeding not related to coronary artery bypass grafting. Major bleeding (life-threatening: fatal or symptomatic intracranial hemorrhage causing decrease in hemoglobin of at least 5 g/dl, requiring surgery, intravenous inotropic or transfusion of 4 or more units; without vital risk: substantial disability, intraocular hemorrhage causing loss of vision or require at least 2 units of transfusion). Minor bleeding: Any other bleeding that caused discontinuation of study medication.

All other medication that the patient is taking is recorded in their medical history and case report file (CRF), as well as any changes they present during the course of the study.

All variables described are introduced by the cardiologist and pharmacist in a database.

### 2.7 Study procedures

Itinerary designed for patient recruitment: The patient is diagnosed with coronary artery disease in the emergency or cardiology departments and is checked to ensure they meet the selection criteria. The patient is informed of the study objectives and the test is performed and informed consent is required. After recording in the CRF that the patient meets the selection criteria, the patient´s clinical situation is reviewed and the corresponding information about the medical history is recorded in the CRF. Every patient will be followed at 1, 3, 6 and 12 months.

### 2.8 Collection and treatment of the biological sample

A saliva sample taken with hyssop is sufficient to obtain a DNA isolation. The sample is sent to Pfizer-University of Granada-Junta de Andalucía Centre for Genomics and Oncological Research (Genyo) where once the genetic material is extracted, the allelic discrimination is carried out by genotyping platform (Single Nucleotide Polymorphism, SNP) Taqman^®^. This technology allows us to make an allelic polymorphism discrimination, indicating the percentage of each allele in the sample. The result is communicated in 24 h to the Pharmacy department at the San Cecilio University Hospital and the clinical pharmacist informs the service of Cardiology of the pharmaceutical recommendation for each patient. At this point the physician prescribes the appropriate treatment for each patient (clopidogrel/prasugrel/ticagrelor). The Pharmacy and cardiology departments do the patient´s follow-up at 1, 3, 6 and 12 months from the start of the treatment and when the patient has a cardiovascular event during follow-up.

Saliva samples are managed through the Biobank, belonging to the National Biobank Network (RD09 / 0076/00148 Project), ensuring the comprehensive treatment of the samples and associated data according to Law 14 / 3 July 2007 biomedical Research, Law 15/1999, of December 13, Protection of Personal data and Law 41/2002 of 14 November, a basic regulatory patient autonomy and rights and obligations regarding information and clinical documentation.

### 2.9 Genotyping

For genotyping, DNA is isolated from saliva sample using standard procedures according to the method detailed in Freeman et al. [2], and in other protocol with some modifications described by Gomez-Martín et al. [3].

The SNPs analyzed in this study are CYP2C19*2 (rs4244285), CYP2C19*3 (rs4986893) and *ABCB1* (rs1045642). These SNPs are genotyped by triplicate using TaqMan^®^ allelic discrimination assay (Life Technologies, Foster City, CA, USA), two replicates are performed in the Genomics Unit of Genyo and a third test is carried out in San Cecilio University Hospital Laboratory.

### 2.10 Withdrawal criteria

The patients can discontinue their participation in the study at any time for any reason if they wish to do so without any consequences. Similarly, the researcher can decide to withdraw a subject from the study if required by the patient´s clinical situation or if the patient does not comply with the protocol. Information about lost patients during the follow-up or leaving the study is collected.

### 2.11 Sample size

The sample size calculation is performed based on the TRITON-TIMI-38 genetic substudy published by Mega et al. [4] in which patients treated with clopidogrel without genetic variation had a cardiovascular event rate of 6.3% compared to those with genotypic variations, reaching a rate event to 13.6%. To achieve 80.0% power to detect differences in the null hypothesis H0: p1=p2 through bilateral chi-square test for two independent samples and considering the significance level is 5%, the study should include at least 263 patients in each group.

### 2.12 Statistic analysis

Firstly, a descriptive analysis of the baseline characteristics of the study subjects is held. Measures of central tendency and dispersion for quantitative variables, and absolute and relative frequencies for qualitative are calculated.

Shapiro–Wilks test is performed to test the normality of the numerical variables, and determine the use of parametric/nonparametric test. To check the homogeneity of the groups a bivariate analysis comparison of the main variables collected between the study groups (genotyping/non genotyping) is performed, the chi-square test or Fisher Pearson for qualitative variables, Student *t*-test or Mann–Whitney test for quantitative are used.

Subsequently, a bivariate analysis is performed to contrast possible differences between groups (Genotyping/non Genotyping) regarding the efficacy and safety (cardiovascular death, nonfatal ACS, urgent revascularization, thrombosis defined stent, ischemic event unrelated to stent thrombosis and bleeding). The same analysis is performed to contrast possible differences between the effectiveness of genotyping previous clopidogrel/ without genotyping.

In addition, a model of multivariate logistic regression is performed for each of the outcome variables, considering as independent variables in the model group assignment (genotyping/non genotyping), those variables with statistical significance in the bivariate analysis, as well as clinically relevant and potential confounders.

The significance level considered to be *p*<0.05. Data are analyzed with statistical software R

### 2.13 Ethical aspects of research

The study is conducted in accordance with the requirements expressed in:

* Law 14/2007 of 3 July, biomedical research.

* Ministerial Order SAS / 3470/2009 establishing guidelines observational post-authorization studies for medicinal products for human use are published.

* Declaration of Helsinki (revised Seoul, October 2008). It defines the principles that must be scrupulously respected by all persons involved in this investigation.

* The treatment, communication and transfer of personal data of all participating subjects shall comply with the provisions of Law 15/1999 of 13 December on protection of personal data.

### 2.14 Information sheet and consent form

Each subject that intends to enter the study is given a written document called "Patient Information Sheet," which contains relevant and necessary information for the patient to decide on their participation in the study (model sheet BIOBANK) (Supplementary data).

The study investigator informs the subject about the voluntary nature of their participation and explains that poses no change in either treatment or medical care compared to those who are not going to participate in the study. The investigator answers the questions and, according to current regulations, obtains the written consent of the subject, or failing orally before independent witnesses of the research team.

It should be clarified completely and unequivocally that the patient is free to refuse to participate in the study and may revoke their consent at any time for any reason without arising for the patient prejudice or be denied treatment or clinical follow-up the researcher. Researchers must retain the signed informed consent on file and must be documented in the medical records of patients.

### 2.15 Confidentiality of data

In order to ensure the confidentiality of the survey data, only the researchers, and the relevant health authorities have access to them.

The data of the researcher and the study are entered into a file, which is treated in accordance with the provisions of the Organic Law 15/1999 of December 13 Protection of Personal Data, exclusively for the development and success of the study.

### 2.16 Study limitations

Selection bias (losses during the follow up).It is an experimental design where the control group is a retrospective group. We reason that this strategy allows us to reduce the recruitment period. To avoid selection bias in the control group, patients are selected based on the inclusion criteria after PCI with stent without being aware of their evolutions during the following period. To prevent the occurrence of confounding biases, the design is pairing strategy, matching the comparison groups regarding the confounders. Likewise, to minimize this bias in the analysis phase, the study results are adjusted in a multivariate model.
