# Supplementary material for: The study protocol for a non-randomized controlled clinical trial using a genotype-guided strategy in a dataset of patients who undergone percutaneous coronary intervention with stent
Source: Data Brief. 2016 Dec 20;10:518–24. doi: 10.1016/j.dib.2016.12.019 (PMC5199189; doi:10.1016/j.dib.2016.12.019)
Supplement: Supplementary file 2 — Supplementary material [file mmc2.zip › supplementary file.doc]

**GRANADA HOSPITAL**

**CARDIOLOGY AND**

**PHARMACY UNITS**

**Patient information brochure**

**WHAT THIS MEANS:**

The departments of Cardiology and Pharmacy from San Cecilio University Hospital, being conscious that a 30% of people who need antiplatelet treatment with Clopidogrel carry a genetic resistence to the drug, they are characterizing and registering this resistence, through the proper genetic study, together with the GENYO research centre.

Genetic studies are procedures used to find differences in the DNA which could provoke CLOPIDOGREL not to be helpful on you.

Because you are a patient with indication of being treated with CLOPIDOGREL because you have been diagnosed with an acute coronary syndrome (Angina pectoris or myocardial infarction) and/or have been treated with a percutaneous coronary intervention and stenting, so you are indicated to use an antiplatelet treatment.

We ask you for your consent to do this genetic study, which will indicate us if we need to customize your antiplatelet treatment to reach the right prevenction effect, and evaluate its benefit.

**HOW WE DO IT:**

The genetic test is done from saliva sample obtained with a swab (something similar to a small lollipop). The follow-up is the usual necessary for the control and monitoring of your disease, including two telephone calls during one year.

**WHICH EFFECTS IT WILL HAVE ON YOU:** none

**BENEFITS:**

Finding many variations on your DNA will help us to modify your treatment to reach the desired effect on the coronary disease prevenction.

**OTHER POSSIBILITIES:**

There are no other posibilities to this genetic test that obtain the same or similar information.

**RISKS:** none.

**OTHER INFORMATION:** We will have your genetic test results before discharge from hospital so your antiplatelet treatment will be guided on the right way.

Your saliva sample will be stored in a Biobank so it can be used by other researchers unless otherwise is declared.

**If you need additional information you can contact us on: –-----**

**INFORMED CONSENT: WRITTEN CONSENT FROM THE PATIENT.**

**Me (Name and Surname):............................................................................................................**

- **I have read the “patient information brochure” given with this informed consent document.**

**I have been able to ask questions about the research “........................................”**

**I have received enough information about the research “.....................................”**

**I have talked with the healthcare professional researcher : ….............................**

**I understand that my participation in this research is voluntary and I can give my consent to this or not to give it.**

- **I have been informed that all the obtained data in this research will be confidential and they will be processed according to the law (Spanish law about personal data protection 15/99).**
- **I have been informed that the data obtained will be used for the research specific purposes .**
- **I would like to be informed about my genetic and personal data obtained during this research, including unexpected breakthroughs, always if this information is neccesary to avoid a big damage on me or my family´s health.**

**YES	NO**

- **I accept the utilization of the sample in future researchs related or not with the disease or process**

**I understand that I can retire from the study:**

- **When I want**
- **Without giving any explanation.**
- **Withput affecting my healthcare.**

**I freely consent my participation on the research: “.............................................................”**

**Patient signature (or legal representative)	Health professional signature**

**Name and surname: ……………….	Name and surname:……………………………**

**Date: ………………………………	Date:……………………….**

Clopidogrel

CYP2C19 and ABCB1

normal function

Prasugrel/Ticagrelor

(CYP 2C19 loss of function and/or ABCB1 TT)

Clopidogrel

Patients with coronary artery disease undergoing PCI with stent and indicating antiplatelet therapy

Genotyping: intervention

No genotyping: retrospective control

- supplementary figure 1
